# Transactions of the State Medical Society

**Published:** 1874-01-01

**Authors:** 


TRANSACTIONS OF THE STATE MEDICAL SOCIETY.—What has become of the Transactions of the Annual Meeting of the Society held in May last? For several years it has been a positive by-law, repeatedly endorsed by the Society, that all papers and reports referred to the Committee of Publication should be placed, by their authors, in the hands of the Permanent Society for the Committee, before the first day of July, and that no delay should be had in publication for papers after that date. Six months have now elapsed since July 1st, 1873, and seven months and a half since the Annual Meeting was held, and no Transactions yet. Some of those who prepared papers, and presented them in due form at the meeting of the Society, are getting a little anxious to see them in print. What is the matter? Who is delinquent? Will the Committee of Publication explain?

